# Macaque monkeys as a non-human primate circadian model

**DOI:** 10.1093/nsr/nwz020

**Published:** 2019-02-05

**Authors:** Han Wang

**Affiliations:** 1 Center for Circadian Clocks, Soochow University, China; 2 Reviewer of NSR

Circadian studies have become one of the prestigious fields in biology, as evidenced by the award of 2017 Nobel Prize in Physiology or Medicine to three pioneers in this field [1]. Even though this Nobel Prize honored the discovery of the molecular genetic basis underlying the fascinating time-keeping mechanisms of living things, it indeed underscored the far-reaching medical implications of the circadian principle. Numerous epidemiological investigations as well as animal model studies revealed that circadian misalignment leads to malfunctions of the body and diseases such as sleep disorders, metabolic diseases, cardiovascular diseases, immune diseases and tumors, birth defects and reproductive problems, and neural and psychiatric diseases [2,3]. However, very little is known about how the circadian system affects these diseases or molecular genetic links between the circadian regulatory components and the pathways leading to them. A good understanding of circadian regulation of these diseases promises to provide better approaches to their prevention, diagnosis and treatment, as an indispensable part of personalized/precision medicine. Probably due to their nocturnal natures, many rodent circadian models of these diseases fail to recapitulate the full spectrum of the clinical symptoms of their corresponding human diseases, particularly neural and psychiatric diseases. Hence, there is a pressing need to develop diurnal animal models that share high similarities in physiology, metabolism and behaviors with humans. In this regard, the cynomolgus monkey (*Macaca fascicularis*), a non-human primate, offers great advantages—especially the transgenic, genome-editing and somatic cell-cloning techniques have already been established for it [4–6]. In these two studies published in NSR Volume 6, Issue 1, the Hung-Chun Chang laboratory teamed up with the Zhen Liu group, and the Qiang Sun group, who recently reported cloning of the cynomolgus monkey with fetal fibroblast cells [5], successfully established the knockout (KO) macaque model for *BMAL1*, a key circadian component, using CRISPR-Cas9 [7] and subsequently cloned the *BMAL1*-modified cynomolgus monkey via somatic cell nuclear transfer (SCNT) [8].

**Figure 1.**
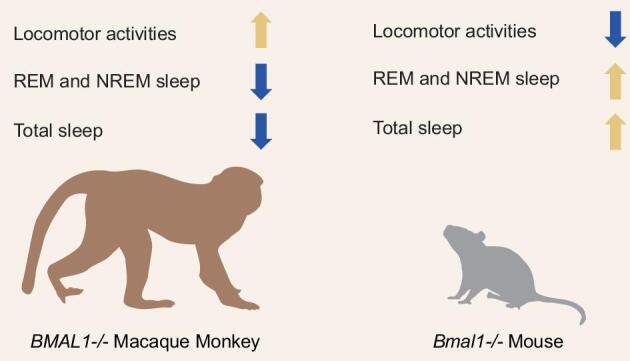
Comparison of locomotor activities, and REM, NREM and total sleep in the *BMAL1–/–* macaque monkey and *Bmal1–/–* mouse. The *BMAL1–/–* macaque monkey and *Bmal1–/–* mouse display completely opposite phenotypes in locomotor activities, and REM, NREM and total sleep, which could shed light on circadian roles in the mysterious and important differences in physiology, metabolism and behaviors of diurnal animals and nocturnal animals.

Qiu *et al.* microinjected two gRNAs targeting exon 13 and one gRNA targeting exon 8 of *BMAL1* into cynomolgus monkey zygotes, transferred 88 microinjected embryos into 31 surrogate recipient monkeys and obtained 8 healthy live births and 2 spontaneously aborted fetuses [7]. Subsequent genotyping identified two *BMAL1* KO male monkeys and one *BMAL1* KO female monkey as well as two *BMAL1* KO dead monkeys [7]. As expected, *BMAL1* expression is abolished in blood cells of these *BMAL1* KO monkeys and its protein is not detectable in various tissues of these two *BMAL1* KO dead monkeys. Further, other key circadian components such as *PER1*, *PER2*, *CRY1* and *CRY2* are all down-regulated in these *BMAL1* KO monkeys [7]. Qiu *et al.* observed elevated locomotor activities in the *BMAL1* KO monkeys during the night-time and reduced REM (rapid eye movement), NREM (non-REM) and total sleep in comparison with wild-type controls [7], which are diametrically opposite to the phenotypes of *Bmal1* KO mice [9,10], indicative of functional differences of diurnal macaque *BMAL1* and nocturnal mouse *Bmal1* (Fig. 1). Intriguingly, the reduced sleep phenotypes are likely associated with much damped plasma melatonin levels without rhythmicity in these *BMAL1* KO monkeys [7]. Through a set of behavioral and electrophysiological assays, Qiu *et al.* clearly demonstrated that *BMAL1* KO monkeys display depression-like, anxiety-like and fear behaviors as well as a schizophrenia-like symptom, which likely resulted from elevated cortisol levels in their plasmas [7]. They then conducted transcriptome analysis of blood samples of the *BMAL1* KO monkey and the wild-type control monkey and revealed up-regulation of inflammatory genes in the *BMAL1* KO monkey, which could contribute to its psychiatric phenotypes [7]. Together, Qiu *et al.* for the first time established a *BMAL1* KO non-human primate model, which will be useful for elucidating the novel aspects of dysrhythmia-derived diseases and developing new therapeutics for them.

The Sun, Liu and Chang team then generated five cloned macaque monkeys with fibroblasts from the 16-month-old young adult *BMAL1* KO monkey using the same SCNT protocol as reported previously [8]. Liu *et al.* transferred 325 SCNT embryos into 65 surrogate monkeys and cloned 5 macaque monkeys with *BMAL1* mutations without mosaicism [8]. Even though the success rate of cloning is still less than 2%, cloning of the *BMAL1* KO monkey is of high significance: first, fibroblasts of a young adult monkey were used; second, transgenics-derived position and dosage effects were avoided; third, the potential genetic mosaicism with genome editing was circumvented; and, finally, it can effectively maintain the *BMAL1* KO monkeys if they have reproductive problems, which likely are derived from loss of BMAL1 [11]. The abilities to clone the macaque monkeys with homogeneous genetic background will facilitate developing non-human primate disease models for unraveling novel pathogenesis mechanisms and developing new therapeutic targets.

These two studies were completed at the Center for Excellence in Brain Science and Intelligence Technology (CEBSIT) and the Institute of Neuroscience (ION), Chinese Academy of Sciences, directed by Mu-ming Pu, who was involved in both studies and has been instrumental in facilitating the development of macaque monkeys as human-disease models. Taken together, these much-anticipated studies usher in a new era of using the macaque monkey as a non-human primate mode for investigating circadian clocks and dysrhythmia-derived diseases.
